# Takotsubo Cardiomyopathy and Catatonia in the Setting of Benzodiazepine Withdrawal

**DOI:** 10.1155/2016/8153487

**Published:** 2016-07-28

**Authors:** Teng J. Peng, Nicholas D. Patchett, Sheilah A. Bernard

**Affiliations:** ^1^Boston University School of Medicine, Boston, MA 02118, USA; ^2^Department of Internal Medicine, Boston Medical Center, Boston, MA 02118, USA

## Abstract

We report two serious and unusual complications of benzodiazepine withdrawal in a single patient: takotsubo cardiomyopathy and catatonia. This 61-year-old female patient was brought to the emergency department with lethargy and within hours had declined into a state of catatonia. Although there was never a complaint of chest pain, ECG showed deep anterior T-wave inversions and cardiac enzymes were elevated. An echocardiogram was consistent with takotsubo cardiomyopathy. She later received 1 mg of midazolam and within minutes had resolution of catatonic symptoms. Careful history revealed that she had omitted her daily dose of lorazepam for 3 days prior to admission. To our knowledge, the case presented herein is the first report of simultaneous catatonia and takotsubo cardiomyopathy in the setting of benzodiazepine withdrawal. The pathogenesis of both conditions is poorly understood but may be indirectly related to the sudden decrease in *γ*-aminobutyric acid (GABA) signaling during benzodiazepine withdrawal.

## 1. Introduction

We report here a patient presenting with two rare diagnoses: benzodiazepine withdrawal catatonia and takotsubo cardiomyopathy. Catatonia is a behavioral syndrome of immobility, rigidity, and mutism and at times restlessness and dysautonomia [1]. Takotsubo cardiomyopathy is a transient left ventricular systolic dysfunction after psychological or physical stress; the condition typically mimics myocardial infarction with apical akinesis, modest elevation in cardiac enzymes, and evolutionary EKG changes (ST elevation or depression or T-wave inversions) [2].

## 2. Case Presentation

A 61-year-old white female was brought to our hospital by family for 8 hours of lethargy and confusion. Medical history was significant for COPD on home oxygen, stage 1A adenocarcinoma of the lung (status post-RLL lobectomy), prior opiate abuse (in stable remission on oral buprenorphine-naloxone), prior alcohol abuse (in stable remission per family), and anxiety treated with lorazepam (2 mg daily). One week prior to presentation, she developed increased dyspnea, cough, subjective fevers, and fatigue. The patient's family administered her usual medications during her acute illness but had omitted her lorazepam for the past 3 days. She became increasingly somnolent and was found on the morning of presentation with a “blank stare” and slurred speech.

On arrival at the hospital, vitals were as follows: temperature 36.8°C, HR 104 beats/min, BP 177/110 mmHg, RR 16, and SpO_2_ 96% on 2 L nasal cannula oxygen. She was moderately somnolent but interacted normally and was oriented to person, place, and time. Within hours, she became persistently disoriented with impaired short-term memory and difficulty following commands. All psychoactive medications were held. After 12 hours, symptoms had progressed to near-complete unresponsiveness, immobility, and periodic agitation. Extensive workup showed no abnormalities to explain symptoms. Blood counts, chemistries, liver enzymes, arterial blood gas, TSH, B12, chest X-ray, and brain MRI were unremarkable. Blood cultures, urine cultures, sputum cultures, and Lyme serology were negative. Urine toxic screen was negative for drugs of abuse, including benzodiazepines. Neurology examined the patient and found no focal neurologic deficits. Based on their exam, they also endorsed low clinical suspicion for nonconvulsive status epilepticus.

Despite absence of chest pain or acute coronary syndrome (ACS) equivalents, admission ECG showed deep anterior T-wave inversions. Troponin I was 0.16 ng/L (normal < 0.013) and rose to 1.37 ng/L over 18 hours. However, ACS treatment was not pursued as the patient was known to have a coronary calcium score of zero and her exercise ECG stress test ten months priorly had shown no inducible ischemia. Urgent TTE was obtained, which revealed hypokinesis and dilation of the left ventricle consistent with takotsubo cardiomyopathy. Left ventricular ejection fraction (LVEF) was 35%, down from 60% ten months priorly.

**Figure 1:**
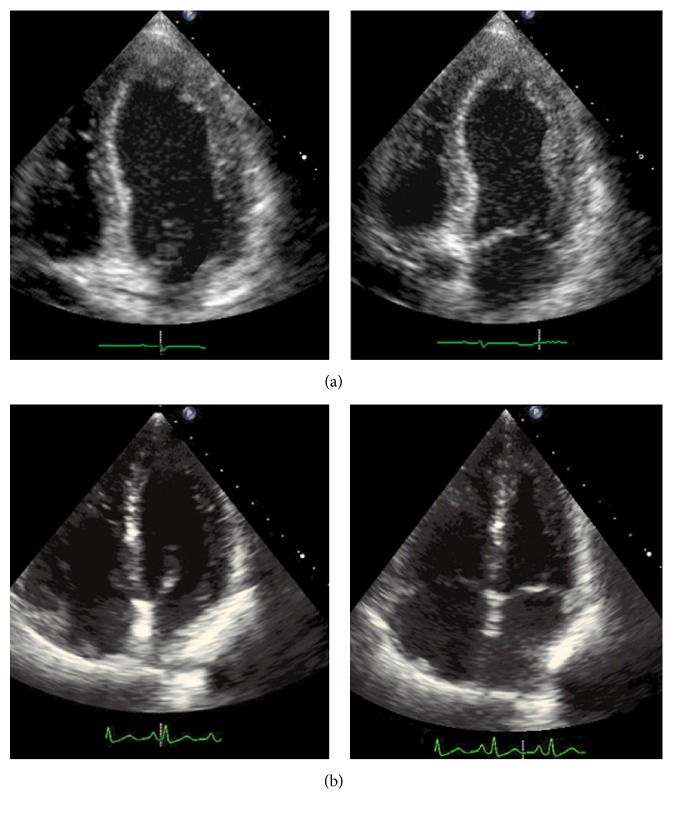
Echocardiogram images obtained during the hospitalization (a) and ten weeks later (b). An apical four-chamber view is shown at end diastole (left) and at end systole (right).

Twenty-four hours after initial presentation, the patient became too restless to allow completion of a routine ECG, so 1 mg of midazolam was given. Within minutes of receiving this medication, she began to talk again and became aware of her surroundings. She was able to recognize her daughter, follow commands, and walk. Scheduled lorazepam was initiated. Within 48 hours, the patient was back to her baseline mental status except for feeling fatigued. She denied chest pain and had no symptoms of congestive heart failure. The patient was discharged on hospital day 3 with a lorazepam taper and metoprolol succinate; TTE 10 weeks later showed reversal of apical remodeling and improvement in LVEF back to 60% (Figure 1).

## 3. Discussion

Catatonia and takotsubo cardiomyopathy are distinct disorders with mechanisms that are not well understood. They have rarely been reported to occur simultaneously [3], and both syndromes have been separately reported in the setting of benzodiazepine withdrawal [4–10]; however, we are not aware of any prior reports of benzodiazepine withdrawal possibly triggering both simultaneously.

Benzodiazepine withdrawal-induced takotsubo has been described twice in published case reports. One [4] described a 49-year-old woman who had her home lorazepam dose (2 mg daily) temporarily withdrawn during a hospital admission. After 28 hours, she began to have symptoms of benzodiazepine withdrawal along with ST-segment elevations on EKG, with a TTE showing apical akinesis and LVEF of 45%. The second [5] described a 65-year-old woman who self-discontinued several long-term psychiatric medications, including a benzodiazepine, and then presented with syncope and a TTE suggestive of takotsubo cardiomyopathy.

Benzodiazepine withdrawal-induced catatonia has been reported several times [6–10]. In these reports, each patient stopped long-standing benzodiazepine therapy, then subsequently became confused and mute with symptoms including muscle rigidity or psychomotor agitation, and then experienced rapid reversal of symptoms after resuming benzodiazepines. None of these reports describe concurrent cardiomyopathy, but several predate the first description of takotsubo cardiomyopathy. Benzodiazepine withdrawal occasionally also precipitates absence status epilepticus, a prolonged seizure that appears similar to catatonia, with symptoms of confusion, disorientation, and a trance-like state [11]. Our patient's symptoms resolved before an EEG could be obtained, so it remains unproven whether her event was ictal or neuropsychiatric in etiology, although our consulting neurologist favored the latter.

While the pathogeneses of catatonia and takotsubo cardiomyopathy are not well understood, their neurohormonal milieu shares common features with benzodiazepine withdrawal. Benzodiazepines potentiate *γ*-aminobutyric acid (GABA) receptor, and thus their withdrawal suddenly decreases the basal inhibitory tone of GABA signaling. Catatonic patients are known to exhibit decreased cortical GABA signaling [12], although this is just one of several postulated mechanisms for catatonia [1]. Takotsubo cardiomyopathy has been reported during withdrawal from multiple other GABA agonists including alcohol [13] and baclofen [14]; therefore, takotsubo may relate at least indirectly to decreased GABA signaling. The direct cause of takotsubo cardiomyopathy is thought to be increased catecholamine release from sympathetic nerves, which induces neurogenic myocardial stunning [15]. In animal models, benzodiazepine withdrawal has been shown to increase catecholamine release in the brain and increases symptoms typical of peripheral sympathetic activity [16].

The case presented herein is the first documented report of simultaneous catatonia and takotsubo cardiomyopathy in the setting of benzodiazepine withdrawal. We propose that the pathophysiology of both conditions may be at least indirectly related to the sudden decrease in central GABAergic tone seen in the setting of benzodiazepine withdrawal, underscoring the “brain-heart” connection in the field of neurocardiology.
